# Development of CD44E/s dual-targeting DNA aptamer as nanoprobe to deliver treatment in hepatocellular carcinoma

**DOI:** 10.7150/ntno.62639

**Published:** 2022-01-01

**Authors:** Cario Wing-Sze Lo, Cecilia Ka Wing Chan, Jianqing Yu, Mian He, Chung Hang Jonathan Choi, James Yun Wong Lau, Nathalie Wong

**Affiliations:** 1 Department of Surgery at Sir Y.K. Pao Center for Cancer, The Chinese University of Hong Kong, Shatin, Hong Kong, China.; 2 Department of Biomedical Engineering, The Chinese University of Hong Kong, Shatin, Hong Kong, China.; 3 Science Research Center, The Seventh Affiliated Hospital of Sun Yat-sen University, Shenzhen 518107, China.; 4 State Key Laboratory of Translational Oncology, The Chinese University of Hong Kong, Shatin, Hong Kong, China.

**Keywords:** CD44, aptamer, nanoprobe, hepatocellular carcinoma, targeted cancer therapy

## Abstract

**Background:** Hepatocellular carcinoma (HCC) is the predominant subtype of liver cancer with an extraordinary high mortality. Resistance to systemic therapy is a major cause of inferior clinical outcome in most patients with HCC. CD44 is a transmembrane cell-surface glycoprotein that is characterized by its variants displaying differential overexpression in human cancers. Aptamers, also known as chemical antibodies, can target cell-surface molecules with high affinity and specificity via structural recognition. Aptamer-mediated drug delivery hence is of high potentials in guiding therapy to improve efficacy.

**Methods:** Variants CD44E and CD44s were studied for HCC relevance by investigating their expressions in primary HCC tumors, adjacent cirrhotic/fibrotic livers and normal livers using junction specific primers in qPCR assay. CD44E/s dual-targeted aptamers were uncovered by integrating loss-gain cell-SELEX and next generation sequencing. Selected aptamers were characterized for binding affinity and specificity, biostability, *in vivo* and *in vitro* cytotoxicity, *in vivo* homing and biodistribution, and ability to deliver 5-FU into targeted cells *in vitro*.

**Results:** Both CD44E and CD44s isoforms showed significant upregulations in HCC tumors with CD44E/s activities promoting cell proliferation and migration. Loss-gain cell-SELEX uncover a CD44E/s dual-targeting aptamer, termed CD44-Apt1. Strong binding of CD44-Apt1 to cell-surface CD44 positive cells but not CD44-negative cells was demonstrated by flow-cytometry. CD44-Apt1 displayed strong affinity to CD44E and CD44s with K_D_ as low as 1 nM but not the hyaluronic acid binding domain of CD44. Confocal imaging of CD44-positive cells stained with fluorescent-labeled CD44-Apt1 showed profound cytoplasmic localization, suggesting efficient cell-penetrating ability. Meanwhile, no apparent staining was observed in CD44-negative cells. CD44-Apt1 when conjugated with inhibitor 5-FU showed efficient guidance of 5-FU into HCC cells that significantly enhanced drug toxicity by more than thousands-fold. Both* in vitro* cell treatment and *in vivo* animal biodistribution indicated that CD44-Apt1 is non-toxic. In HCC xenograft model, CD44-Apt1 efficiently homed to tumor xenografts in a CD44 expression-dependent manner.

**Conclusion:** Novel discovery of aptamer CD44-Apt1 that can bind both CD44E and CD44s illustrates high potential as nanoprobe to deliver anti-cancer therapeutics.

## Introduction

Hepatocellular carcinoma (HCC) accounts for 90% of all primary liver cancer and is the third leading cause of cancer mortalities worldwide with a 5-year overall survival rate of 10% 1. The dismal clinical outcome is largely attributed to ineffective therapeutic interventions in advanced stage HCC patients. Targeted nanoscale-based drug delivery system in guiding therapeutics into targeted tumor cells is expected to increase intracellular drug loading and improve therapeutic efficacy. Aptamers, also known as chemical antibody, are single-stranded synthetic DNA or RNA oligonucleotides that can fold into stable, unique three-dimensional structure for specific docking onto targeted molecules via structural recognition with high affinity and dissociation constants as low as pico- to nano-molar range 2. Compared to protein antibodies, aptamer is non-immunogenic in nature. It could be denatured at high temperature for a brief period then fold back into functional three-dimensional structure and is thus incredibly thermal stable. With its small size, usually small than 50 kDa, it could be efficiently internalized into cells upon binding to specific cell-surface proteins via clathrin- and caveolae-dependent endocytosis 3, and have been shown to demonstrate strong cell penetrating ability 4. Due to its nucleic acid in nature, aptamers can be easily chemically modified with high plasticity 30. Aptamer-based therapeutics and diagnostics have hence attracted much attention in clinical applications and cancer research, for instance linking with nanoparticles or reporter molecules for diagnostic *in vivo* imaging 5 , or itself acting as antagonist or conjugated with anti-cancer drug for therapeutic purposes 6, 7. Nevertheless, identifying specific cell-surface proteins is vital for developing high affinity aptamer binders.

**Figure 1:**
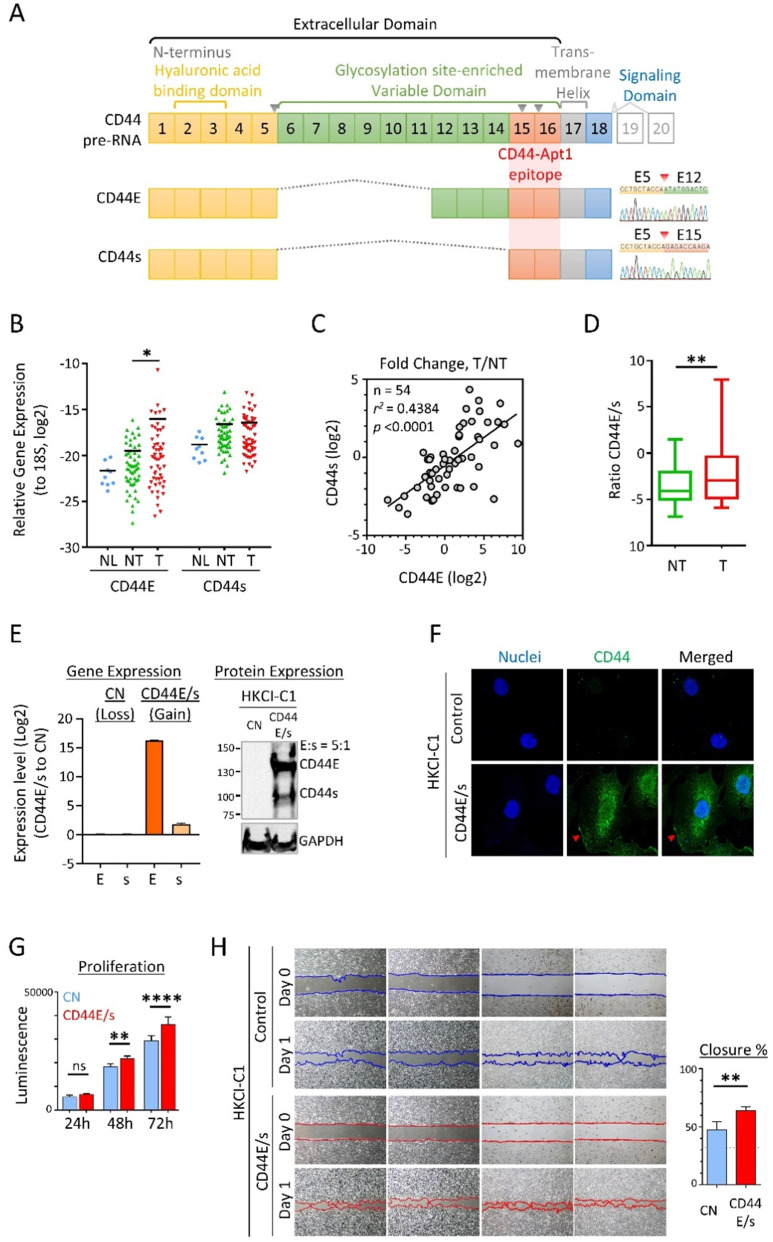
** (A)** Gene structure and protein domain arrangement of CD44 and two prevalent isoforms CD44E and CD44s in HCC. CD44 consists of 20 exons. Exons 1-5 (yellow box) and 15-18 encode the standard isoform CD44s and are conserved in both isoforms. Exons 1-15 depict the extracellular domain with Exons 2-3 for the hyaluronan binding domain and Exons 6-14 (green box) for the variant domain harboring abundant glycosylation sites. Exon 17 (grey box) is for transmembrane helical domain, while Exon 18 (blue box) encodes the intracellular signaling transduction domain. Exons 19 and 20 (grey open box) are mutually exclusive exons. Alternative splicing between Exons 5 and 15 resulted in the CD44E isoform. Supporting sequencing traces for the detection of CD44s and CD44E isoforms in HCC are provided. Epitope region bound by CD44-Apt1 is also suggested. Grey inverse triangles indicate reported MMP cleavage sites on CD44 protein. **(B)** Relative expression of CD44E and CD44s genes in nine human normal liver tissues (NL) and fifty-four pairs of HCC tumoral tissues (T) and adjacent non-tumoral tissues (NT). Expression is normalized to S18 ribosomal RNA. **(C)** Correlation in fold change (T/NT) between CD44s and CD44E gene expression. **(D)** Ratio of CD44E to CD44s in TN and T in HCC. **(E)** Establishment of CD44E- and CD44s-dual-overexpressed HCC stable cell line (CD44E/s). Gene expression of CD44E (E) and CD44s (s) in HKCI-C1 vector control (CN) and CD44E/s cells were determined by real-time quantitative PCR and compared between the two cell lines. For CD44 protein expression, 20 ug of total protein of either CN or CD44E/s cell lysates were resolved on a 5-12% gradient acrylamide gel. Membrane with proteins transferred from the gel was blotted with anti-CD44 or anti-GAPDH antibodies. GAPDH protein was used as an endogenous loading control. Relative quantity of CD44E to CD44s protein (E:s) was determined based on the their protein band intensities. **(F)** Localization of CD44 in the HKCI-C1 cells by confocal imaging. CD44 protein (green) in the CN and CD44E/s cells was stained with primary CD44 antibody and Alexa fluor-488 labeled secondary antibody. Nuclei were stained with Hoechst (blue). The stained cells were imaged using confocal microscope under 100X magnification. Red arrows point to the membrane edge.** (G)** Proliferation potential of HKCI-C1 cells. The CN and CD44E/s cells were seeded at a density of 1000 per well. Cell viability was measured after 24, 48 and 72 hours using Celltiter-Glo. Luminescence signal plotted, mean ± SEM (n=8). Comparisons between CN and CD44E/s were done using 2-way ANOVA test with Bonferroni correction. **(H)** Migratory phenotype. HKCI-C1 CD44E/s and CN cells were seeded on two-chamber culture dish. Insert between the two chambers was removed at Hour 0. Bright field images were taken at Hour 0 and 13. Closure percent was calculated as the percentage of gap area that occupied by the cells between Day 0 and Day 1 (mean ± SD, n=4). Measurements were compared using paired t-test. *p < 0.05, **p < 0.01, ****p < 0.0001.

Our group has previously transcriptome profiled HCC for full-length mRNA by long-read sequencing and sketched a comprehensive map of alternative spliced variants of HCC. A series of HCC-enriched alternative spliced isoforms, such as CD44 variants CD44E (also called CD44v8-10) and CD44s (also called CD44v1) 8. CD44 is a family of cell-surface glycoproteins, with each member containing an extracellular domain at its amino terminal that consists of a hyaluronic acid binding domain and a variable-length hyper-glycosylation site-harboring region 9, followed by a transmembrane helical domain, and an intracellular signaling transduction domain 10, 11. In human, the CD44 gene comprises about 20 exons (**Figure 1A**). The canonical CD44 isoform, CD44s, integrates Exon 1-5 and Exon 15-18 that encode the hyaluronic acid binding domain (HABD) and the transmembrane and intracellular domains, respectively, serves as a common backbone for other CD44 splice variants. For instance, CD44E is formed by the backbone exons of CD44s plus Exon 12-14, and therefore shares domain regions with CD44s. Cumulative evidence showed both CD44E and CD44s to play pivotal roles in cancer biology, including resistance to ROS-mediated apoptosis and the process of epithelial-to-mesenchymal transition (EMT) 12-14. In addition to their broad functionalities, the cell membrane nature of both variant proteins highlights a promising feature that can be exploited to generate cell-surface-specific aptamers to direct therapy.

In this study, we first assessed the pattern of CD44E and CD44s upregulations in an independent HCC patient cohort. We then developed a CD44E/s expressing (gain) and non-expressing (loss) cell line with the ratio of variant proteins representative of primary HCC tumors (5 CD44E: 1 CD44s). Functions of CD44E/s isoforms in these HCC cells were also examined. Taking into consideration of the probable posttranslational modifications under the HCC cellular context, discovery of CD44E/s aptamers was conducted using live HCC cells by the technique of loss-gain Cell-based Systematic Evolution of Ligands by Exponential enrichment (cell-SELEX) 15, 16. We speculated that drug efficacy could be enhanced if delivered by a CD44E/s aptamer via endocytosis after binding to tumor cell membrane 17. The identified CD44E/s aptamer was hence characterized for binding specificity and affinity, cell-penetrating ability and subcellular localization, biostability against biological medium, *in vitro* and *in vivo* cytotoxicity, ability to guiding drug into cells for enhancement of drug sensitivity, as well as *in vivo* homing ability to HCC and xenograft biodistribution.

## Materials

### Patients Specimens

Paired HCC tumor and adjacent nontumoral liver tissues were collected from patients who underwent curative surgery at Prince of Wales Hospital, Hong Kong. Human sample collection protocol was approved by The Joint Chinese University of Hong Kong - Hospital Authority New Territories East Cluster Clinical Research Ethics Committee (CREC Ref. No 2020.420). Informed consent was obtained from each patient recruited. Diagnosis of HCC was confirmed by histology.

### CD44E and CD44s expressions in HCC

Total RNA was extracted using RNeasy Mini Kit (Qiagen) with on-column DNase-I digestion. First-strand cDNA was synthesized using the Superscript^TM^ III First-Strand Synthesis kit (Invitrogen). The cDNA was purified using PCR purification kit (Tingan) and eluted in DNAse- and RNAse-free water. Real-time quantitative PCR (qPCR) was performed with each 20-µl reaction mix containing the purified cDNA (equivalent to 10 ng of total RNA), variant-specific primers at a final concentration of 250 nM, and 5 µl of POWER SYBR green master mix (Invitrogen). The reactions were ran using the QuantiStudio 7 Flex Real-time PCR system (ABI). Data were analyzed using the QuantiStudio 7 software.

### Cell lines and cell cultures

An HCC cell line, HKCI-C1, was established from patient specimen according to standard protocols and maintained in AIM-V medium supplemented with 10% fetal bovine serum (FBS, Thermo Fisher Scientific). Hep3B, Huh7, and MIHA were commercially obtained (ATCC) and were maintained in Dulbecco's modified Eagle's medium supplemented with 10% FBS (Thermo Fisher Scientific).

To transduce stable expression of CD44E/s proteins in HKCI-C1 and Hep3B cells, standard protocol using retrovirus infection was used. Open reading frames of CD44E and CD44s were cloned into a mammalian vector pQCXIP (Addgene) separately. For generating HKCI-C1 CD44E/s or vector control cells, plasmids were transient transfected into Platinum-A retroviral packaging cells (Cellbiolabs) using Opti-MEM (Gibco) using Lipofectamine 3000 (Invitrogen) for retrovirus package. For Hep3B CD44E and CD44s cells, CD44E or CD44s plasmids were transient transfected into Platinum-A cells using the same method. Medium with lipofectamine and plasmids were replaced with complete medium after 8 hours of transfection. After 24 hours, the media with virus were collected, and fresh media were added to the cells for another 24-hour incubation. For both batches, the media were filtered using 0.45 µm filter. The filtered media were applied to either HKCI-C1 or Hep3B cells for infection for two days. After infection, the cells were selected against puromycin (Thermo Fisher Scientific).

### Cell proliferation and migration

For proliferation, HKCI-C1 vector control or CD44E/s cells were seeded on 96-well plate at cell density of 1000 cells per well and cultured in complete medium for 24, 48, or 72 hours. At each end point, cell viability was accessed using Cell-titer Glo (Promega). For migration, HKCI-C1 vector control or CD44E/s cells were seeded on µ-Dish 35mm with 2-well culture-insert at a cell density of 2500 cells per well. When the confluency reaches 95%, the insert was removed to start the assay. Bright field images were taken at hour 0 and hour 13. Images were analyzed using Image J 18.

### Western blotting

To obtain protein lysate, cells were lysed in 1X RIPA lysis buffer (Abcam) with supplement of 0.1% Triton-X 100 and 1X protease inhibitor cocktail (Roche), followed by incubation on ice for 15 minutes and subsequent sonication using conditions of 1 sec ON (40% amplitude) and 1 sec OFF for two cycles. The lysate was centrifuged at 15000g at 4 °C for 15 min, and supernatant was collected. Total protein concentration was measured using Pierce^TM^ BCA kit (Thermo Fisher Scientific) with bovine serum albumin (BSA) as standard. For western blotting, protein lysate was mixed with 1X sample buffer (Invitrogen) that contains 10% of β-mercaptoethanol and denatured at 95 °C for 10 min followed by centrifugation at 15000g at 4 °C for 10 min. Supernatant was loaded on 5-12% gradient polyacrylamide gel and resolved by SDS-PAGE. Proteins on gel were transferred to PVDF membrane. Antibodies against CD44 (ab243894, Abcam) and GAPDH (Cell Signaling) were used.

### Cell-SELEX

Aptamer library with 40 nucleotide random sequences flanking by 18 nucleotide upstream and downstream known-sequence primers was commercially synthesized (IDT oligo). Thioate modified, fluorescent labeled, and 5-FU conjugated aptamers were in-house synthesized by solid phase synthesis. Cell-SELEX protocol was adopted from Sefah *et al* 15. Prior to start, washing buffer that contains 1 L of DPBS with 4.5 g of glucose, and 5 ml of MgCl_2_ 1M, and binding buffer that contains 1 L of DPBS with 4.5 g of glucose, 100 mg yeast tRNA, 1g BSA, and 5 ml of MgCl_2_ 1M, were prepared.

In the first cycle, functional aptamer library was prepared in 1 ml of 1X binding buffer to a final concentration of 20 µM, heated at 95°C for 5 min and left at room temperature for 1 hour. The library was applied to HKCI-C1 CD44E/s cells that were at 95% confluency in a 15-cm dish. These cells were pre-washed with 1X washing buffer twice. After 1 hour of incubation at room temperature with gentle rocking, the unbound aptamers were removed, and cells were washed with washing buffer twice and collected in 1 ml of DNase-free water using scraper. Cell suspension was heated at 95 °C for 10 min and centrifuged at 15000g at 4 °C for 10 min. Supernatant obtained was mixed with AmpliTaq Gold^TM^ 360 Master Mix (Thermo Fisher Scientific), biotin-labeled reverse primer and Cy5-labeled forwards primer to retrieve the bound aptamers by PCR. To prevent over-amplification, the aptamers were recovered by two rounds of PCR. In the first round, only 6 cycles of PCR were performed. The number of cycles in the second PCR was determined by cycling optimization. For optimization, ten tubes of 20-µl second PCR mixture each contains 1/20X of first PCR products, aptamer primers and master mix, were prepared. Cycling condition was basically the same as that for the first PCR but differing in the number of cycles. One tube of PCR product was collected after every alternative cycle of 10 to 28. The collected second PCR products were resolved by 3% agarose gel. The cycle number with maximum amount of single product was chosen.

A total of 6-ml second PCR was prepared. The PCR products were isolated using Streptavidin magnetic beads (Invitrogen) and washed with washing buffer three times and water one time. The Cy5-labeled forwards-strands were separated from the biotin-labeled reverse strands by incubating with 200 mM of NaOH for 2 min at room temperature. The isolated single-stranded aptamers were purified using oligo purification kit (Tiangen). Purified aptamers were then applied to the next cycle of selection. In subsequent selection cycles, the concentration of aptamer pool was adjusted to 200 nM. Only gain selection, or so call positive selection, was performed in the first 2 cell-SELEX cycles. Starting from Cycle 3, a loss selection, or so call negative selection was applied before the positive selection. The purified aptamer pool from the last cycle was firstly applied to the negative cells, i.e., HKCI-C1 vector-control cells. After 1 hour of incubation, aptamer supernatant from the negative cells was collected and applied directly to positive cells, i.e., HKCI-C1 CD44E/s cells for positive selection. For identification of CD44E/s aptamers, a total of eleven iterations were performed. Aptamer pools of alternative cycles were sequenced by next generation sequencing. Obtained sequences were analyzed using FASTAptamer 19 for CD44E/s aptamer identifications.

### Flow cytometric analysis of aptamer-bound cells

Adherent HCC cells were detached by incubating with 10 mM of EDTA at 37 °C until 95% of cells detached. Cells were washed with 1X PBS twice and blocked with 20% FBS in 1X PBS for 20 min. The FBS-blocked cells were incubated with 100 nM of fluorescent-labeled CD44-Apt1 at room temperature with gentle agitation for 1 hour. After incubation, cells were collected by centrifugation at 1200 g at 4 °C for 5 min and washed with 1X PBS twice. The washed cells were resuspended in 1% PFA and passed through 70 µm cell strainer and keep on ice. Fixed cells were analyzed using flow cytometer Cell Analyzer BD FACSDiva (BD). Collected data were processed using the software FlowJo (BD).

### Immunofluorescence staining and imaging

For anti-CD44 staining, cells were seeded on µ-Slide 8-well (Ibidi) at a cell density of 20,000 and cultured for 24 hours. The cells were washed with 1X PBS twice, then fixed with 4% paraformaldehyde at room temperature for 30 min. The fixed cells then were washed with 1X PBS twice and permeabilized using 0.1% Triton-X 100 and incubated at room temperature for 10 min. Cells were washed again with 1X PBS twice and blocked with 5% BSA overnight at 4 °C. The blocked cells then were stained with CD44 primary monoclonal antibody (ab243894, Abcam) and Goat anti-Rabbit IgG secondary antibody with conjugation of Alexa Fluor Plus 488 (A32766, Thermo fisher). Nuclei were stained with Hoechst. Cells were imaged using the Confocal microscopy LSM 880 Laser Confocal Microscope (Carl Zeiss).

For live cell staining using CD44-Apt1, cells were seeded on µ-Slide 8-well (Ibidi) at a cell density of 20,000 and cultured for 24 hours. Cy3-conjugated CD44-Apt1 was added to cells at a final concentration of 500 nM. The cells were further cultured at 37 °C for 24 hours and then washed with 1X PBS for 3 times and fixed in 4% PFA for 30 min. Nuclei were stained with Hoechst. Cells were imaged using the Confocal microscopy LSM 880 Laser Confocal Microscope (Carl Zeiss).

### Aptamer stability test

Human serums were obtained from two healthy volunteers. One nmol of phosphorothioate-modified CD44-Apt1 was added to 90% freshly obtained human serum and incubated for 0 to 96 hours. After incubation, the aptamers were linearized using NaOH at a final concentration of 200 mM and then resolved in 3% agarose gel by electrophoresis.

### Determination of Aptamer binding affinity by ELISA assay

Monoclonal anti-CD44 antibody (ab243894, Abcam) or Anti-His antibody (2365S, Cell Signaling) was diluted in 1X bicarbonate buffer with a dilution ratio of 1:3000. The antibody was coated on an ELISA plate by adding 50 µl of diluted antibody per well and incubating overnight at 4 °C. On the next day, antibodies in the wells were removed, and the wells with coated antibodies were washed with 1X PBST (1X PBS supplemented with 1% Tween-20) three times with 3 min incubation time between washes. Protein lysate of CD44s- or CD44E-OE cells or His-tagged HABD protein were diluted with 1X PBS and applied to the CD44 antibody- or Anti-His tag antibody-coated wells and incubated for 2 hours at room temperature with 200 rpm of agitation. After incubation, the unbound proteins were washed off using 1X PBST. Biotin-labeled CD44-Apt1 was serially diluted from 10 µM to 6 pM in 1X binding buffer. The diluted biotin-labeled CD44-Apt1was applied to the protein-bound wells in quadruplicate per condition and incubated for 1 hour at room temperature with 200 rpm of agitation. The unbound aptamer was washed off using 1X PBST. Fifty microliters of Pre-diluted High Sensitivity Streptavidin-HRP (Pierce) were added to each treated well and incubated for 1 hour at room temperature with 200 rpm of agitation. The treated wells were washed with 1X PBST. A hundred microliters of 1-Step^TM^ TMB-ELISA Substrate Solution (Thermofisher) were added to each treated well and incubated until a noticeable trend of color change across aptamer concentrations. Reactions were stopped by adding 100 µl of 3M of HCl per treated well. Absorbance signal was detected at 450 nm using SpectraMax iD3 (Molecular Devices).

### *In vivo* homing of CD44-Apt1 to HCC xenografts

HCC xenografts were developed using Hep3B vector control, CD44E, and CD44s cells (Animal Experimentation Ethics Ref No. 20-198-AOE). For each HCC xenograft, 5 x 10^6^ of cells were resuspended in 100 µl of matrix gel and subcutaneously injected on the right flank of a nude mouse. When the xenograft reached a size of about 200 mm^3^, Cy3-labeled CD44-Apt1 or binding buffer vehicle were intravenously injected via caudal vein and incubated for 72 hours before sacrifice. Tumor and major organs were harvested and imaged using ChemiDoc^TM^ touch imaging system (BioRad). The tumor size was calculated based on the equation of volume = length x width^2^/2.

### *In vivo* cytotoxicity test of CD44-Apt1

A total of 250 pmol of CD44-Apt1 in 100 µl of binding buffer or 100 µl of binding buffer was intravenously injected in nude mice. After 3 days, mice were sacrificed. Organs, including brain, heart, lung, liver, spleen, stomach, intestine, and kidney were harvested and fixed with 4% PFA at 4 °C overnight and processed for H&E staining on the next day. Stained slides were imaged using ECLIPSE Ni-U microscope (Nikon).

### *In vitro* Drug efficacy test of CD44-Apt1-5FU and 5FU on HCC cells

HCC cells were seeded on 384-well white plate at a cell density of 750 cells in 50 µl of complete medium per well. Treatment was performed one day after seeding. CD44-Apt1 with or without conjugation of 5-FU were serially diluted using binding buffer at 5X concentration first and diluted to 1X with serum-free medium before adding to the cells. 5-FU was serially diluted using DMSO to 33X first and further to 1X with serum-free medium before addition to cells. The cells were incubated with CD44-Apt1, CD44-Apt1-5FU or 5FU for 72 hours. Cell viability was determined using Celltiter-Glo (Promega).

### *In vivo* growth inhibition effect of CD44-Apt1-5FU and 5FU on HCC xenografts

HCC xenografts were developed using Hep3B vector control, CD44E, and CD44s cells. For each HCC xenograft, 3 × 10^6^ of cells were resuspended in 50 µl of matrix gel and subcutaneously injected into the right flank of a nude mouse. When the xenograft reached a size of about 100 mm^3^, mice were divided into four aliquots based on tumor size. Mice groups were treated with either CD44-Apt1-5FU (250 pmol or 1nmol) or equivalent amount of 5FU by intravenously injection through the tail vein for 8 consecutive days. The xenografts were allowed to further grow for 2 to 6 days until sacrifice. Width and length of the xenografts and the body weight of the mice were measured across the whole experiment since the day before treatment to the sacrifice day. The tumor size was calculated based on the formula of volume = length × width^2^/2. Weights of the harvested tumors were measured on the sacrifice day.

## Results

### Common upregulations of CD44E and CD44s in HCC tumors

Expressions of CD44E and CD44s in HCC were evaluated by exon-flanking quantitative PCR, and confirmatory sequencing to establish specificity (**Figure 1A**). In 54 pairs of primary HCC tumors and adjacent non-tumoral tissues examined, as well as reference normal liver tissue samples (n=9), expressions of CD44E and CD44s showed frequent upregulations in tumors. Compared to the normal liver tissue reference pool, both isoforms showed an increasing trend of expressions with a median 4.2-fold (CD44E) and ~2-fold (CD44s) upregulations detected in tumors (**Figure 1B**). A moderate positive correlation between CD44E and CD44s expressed observed in tumors suggested a common co-expression of these two variants in HCC (**Figure 1C**). Interesting to note, the expressed ratio of CD44E to CD44s is 5.2-fold in tumors while only 0.3-fold in non-tumoral liver tissues, which would imply a differential alternative splicing pattern of isoform switch between tumoral and non-tumoral stage (**Figure 1D**). When compared to adjacent non-tumoral liver tissues, 50% HCC tumors showed upregulation of CD44E with fold change greater than 2. Overall, up to 61.1% of HCC tumors displayed increased expression of either one of CD44E or CD44s (fold change > median).

### CD44E/s dual-targeting aptamers

We took advantage of the cell membrane distribution of CD44 variants for aptamer discoveries and developed an HCC cell model (HKCI-C1) with both CD44E and CD44s over-expressed at a ratio mimicry in primary HCC tumor of 5:1 (**Figure 1E**). Immunofluorescent staining supported the cell-surface localization and internalization of overexpressed CD44E/s (**Figure 1F**). We also examined the biological effects of CD44E/s, which showed an increased cell viability and migratory ability of CD44E/s cells compared to vector-control isogenic line (**Figure 1H & G**).

**Figure 2:**
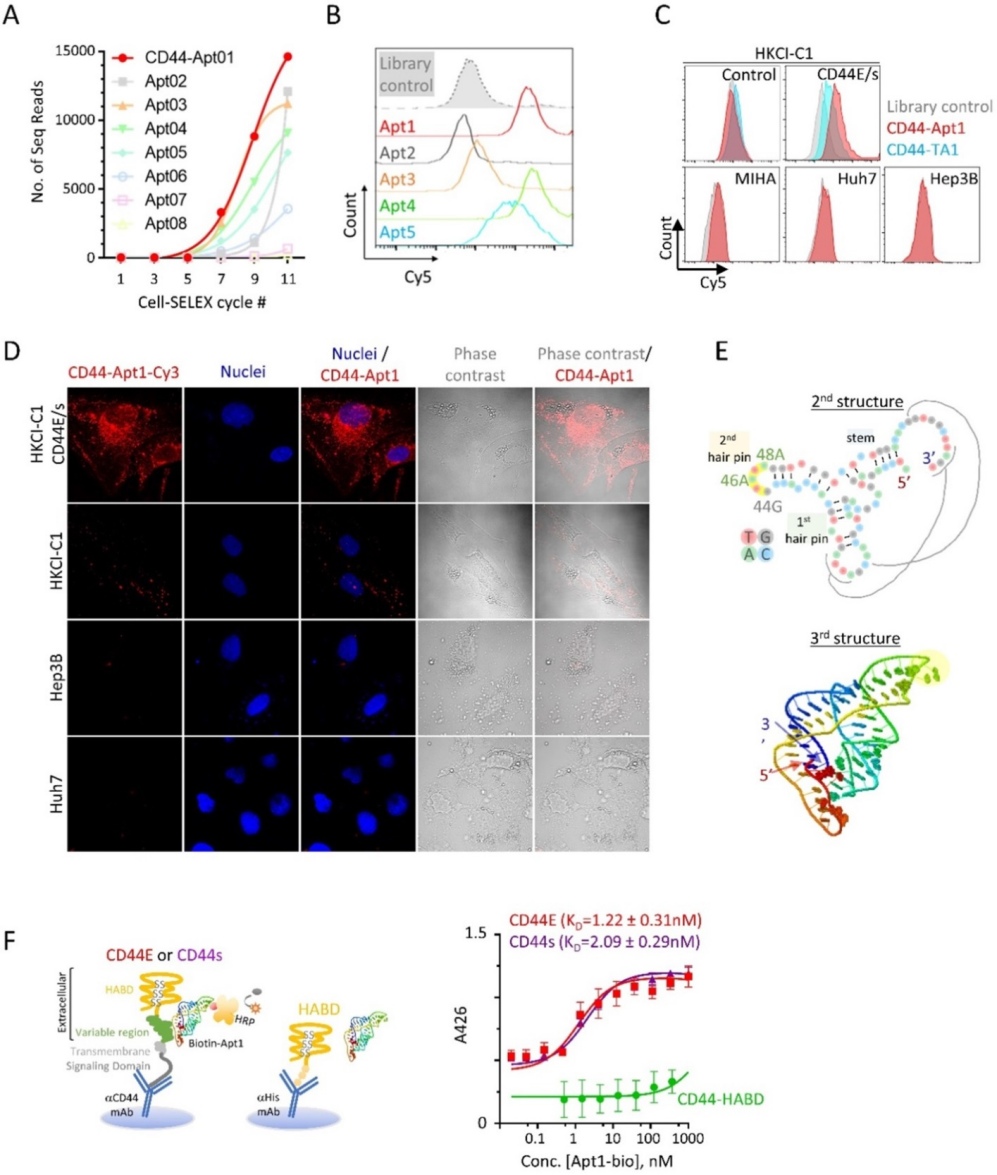
** Identification and characterization of CD44E/s aptamers. (A)** Enrichment of CD44-specific aptamer sequences by the technique of loss-gain CELL-SELEX. Aptamer pools from alternative cycles during Cycle 1 to 11 were sequenced by next-generation sequencing. For the top eight abundant aptamers, named CD44-Apt1 to -Apt8, number of sequencing reads in different cycles were plotted to illustrate their gradual enrichment across the SELEX process. **(B)** Flow cytometric analysis of HKCI-C1 CD44E/s cells after incubation with Apt-1 to -Apt5 and library control. These 5 aptamers and aptamer library were conjugated with Cy5 fluorescence and incubated with HKCI-C1 CD44E/s cells at 200nM for 1 hour at room temperature with agitation before analyzing by flow-cytometry. Fluorescence intensity of Cy5 was plot in the histograms. **(C)** Flow cytometric analysis of various human liver cells after incubation with Cy5-labelled CD44-Apt1 aptamer. HKCI-C1 cells, including vector control and CD44E/s, and a normal liver cell line MIHA, and two CD44-negative HCC cell lines, Huh7 and Hep3B cell lines were stained with Cy5-labeled CD44-Apt1 (red), Cy5-labeled CD44 aptamer TA1 (blue) 20 or library control (grey) and analyzed by flow cytometry. Fluorescence intensity of Cy5 was plot in the histograms. **(D)** Confocal fluorescence microscopy of HCC cells for localization of CD44-Apt1. HKCI-C1 vector control and CD44E/s cells, Hep3B, and Huh7 were live-stained with Cy3-labeled CD44-Apt1 (red), and then fixed and imaged by confocal microscopy. Nuclei were stained using Hoechst (blue). Phase-contrast images were taken to illustrate the outline of cells. **(E)** Predicted secondary and tertiary structure of CD44-Apt1. Black lines indicate the nucleotide interaction between the loop of first hairpin and the sequence at the most 3' end when Apt1 folded into 3D structure. **(F)** Epitope mapping and binding affinity determination of CD44-Apt1 using sandwich ELISA. CD44E and CD44s proteins were captured on the bottom of ELISA plate using anti-CD44 antibody that targets COOH-terminal intracellular domain and remains the N-terminal extracellular domain intact for aptamer binding. His-tagged recombinant CD44 HABD protein was immobilized on ELISA plate using His-tag antibody. The captured CD44 proteins were incubated with various concentrations of biotin-labeled CD44-Apt1 (5 pM - 10 µM). Dissociation constant, K_D_ was calculated based on the absorbance values from replicates (n=3) using the one-site binding model for non-linear regression in GraphPad.

To identify aptamers targeting CD44E/s, we utilized the Loss-Gain cell-SELEX technique 15. In the gain step, CD44E/s positive cells were used to capture both CD44E/s aptamers. Non-CD44 binders could also be captured through binding to other receptors on HKCI-C1 cells, and these were subsequently removed in the loss step, where HKCI-C1 vector control cells were deployed. We repeated this loss-gain cell-SELEX for eleven cycles to enrich the CD44-specific aptamers gradually and exponentially from cycle to cycle. The aptamer pools from alternative cycles starting from the first cycle were later sequenced by the next-generation sequencing. Based on the sequencing results, a total of five aptamers, named CD44-Apt1 to -Apt5, were ascendingly enriched across cycles (**Figure 2A**). These five aptamers were individually synthesized and conjugated with Cy5-flurophore for flow cytometric analysis in CD44E/s cells. Compared to library control, CD44E/s cells stained with the aptamers, other than CD44-Apt2, accumulated Cy5-signal that resulted in a signal shift (**Figure 2B** and **Table 1**). Since CD44-Apt1 was the most abundant aptamer after 11 cycles of selections and showed strongest binding among all aptamers to CD44E/s cells, CD44-Apt1 was chosen for further analysis.

We next assessed the specificity of CD44-Apt1 binding. The HKCI-C1 vector control cells and CD44E/s cells, and three cell lines with negligible CD44 expression, including a normal liver cell line MIHA and two live cancer cell lines Hep3B and Huh7, were lively stained with Cy5-CD44-Apt1 or aptamer library. Flow cytometric analysis of the stained HKCI-C1 CD44E/s cells revealed a shift in Cy5 signal between Cy5-CD44-Apt1 and library, suggesting binding of CD44-Apt1 to the overexpressed CD44E/s on the cells (**Figure 2C**). In contrary, with the HKCI-C1 vector control cells and the three cell lines, flow cytometric analysis of stained cells showed no shift in Cy5 signal between Cy5-CD44-Apt1 and library, suggesting absence of non-specific binding (**Figure 2C**). In another experiment, a Cy3-labeled CD44-Apt1 and nuclei dye Hoechst were used to stain cells, including CD44E/s, vector control, Hep3B, and Huh7. The cells were then visualized by immunofluorescence confocal imaging (**Figure 2D**). In CD44E/s cells, CD44-Apt1 showed apparent localization on the cell membrane, and also into the cytoplasmic and nuclear compartments. Conversely, HKCI-C1 vector control cells displayed minimal CD44-Apt1 detection. Consistent with flow cytometry analysis, CD44-Apt1 binding was not observed in CD44-negative Hep3B and Huh7 cells. Our flow cytometry and confocal imaging suggested high specificity of CD44-Apt1 towards CD44E/s variant proteins.

Based on the sequence of CD44-Apt1 (**Table 1**), secondary structure prediction suggested CD44-Apt1 to form two hair pins and one stem with a minimum free energy of -19.7kcal/mol (**Figure 2E**). This secondary structure was used to simulate the tertiary folding of CD44-Apt1. The tertiary folding revealed an interaction of the loop region of the first hairpin and distal sequence at 3' end. This would suggest the loop of second hairpin from nucleotide 44G to 48A is completely exposed and is an ideal region for biotin labelling in our subsequent binding affinity experiments. Although the 14-base sequence at the most 3' end seems like a free fragment based on the secondary structure prediction, this sequence buries at the core of the whole aptamer based on the tertiary folding prediction and it interacts with bases in the loop of first hairpin. We therefore did not trim the sequence of CD44-Apt1.

We next determined the major epitope in the CD44 protein that is recognized by CD44-Apt1. CD44 protein consists of an extracellular domain, a transmembrane helical domain, and an intracellular signaling transmission domain (**Figure 1A & 2F**). The extracellular domain contains a hyaluronic acid binding domain (HABD) at its distal N-terminus and a glycosylation-site enriched variable region, the length of which varies in different spliced variants. Titration sandwich enzyme-linked immunosorbent assay (ELISA) was employed to map the epitope for CD44-Apt1. CD44E and CD44s proteins were captured at their intracellular signaling domain by a monoclonal CD44 antibody that was immobilized on a support, while a recombinant protein of His-tagged CD44-HABD was captured via His-tag antibody on another support. The CD44-Apt1 was labelled with biotin at the nucleotide 44G, 46A and 48A of the loop in the second hairpin (**Figure 2E**). A series of titrated biotin-labeled CD44-Apt1 was applied to the captured proteins. The amounts of biotin-CD44-Apt1 bound to the captured proteins were evaluated using streptavidin conjugated horseradish peroxidase (HRP) and TMB substrate. CD44-Apt1 showed strong affinity towards CD44E and CD44s proteins with an equilibrium dissociation constant K_D_ of 1.22 ± 0.31 nM and 2.09 ± 0.29 nM, respectively, whereas it was hardly associated with CD44-HABD protein fragment (**Figure 2F**). Since only the extracellular domain of CD44 was exposed when performing cell-SELEX, and that CD44-Apt1 showed only weak binding affinity to CD44-HABD, the major epitope of this aptamer is expected to be within the domain region encoded by Exons 15 to 16 (**Figure 1A**).

### CD44-Apt1 guiding dramatically increases anti-cancer drug sensitivity of HCC cells

**Figure 3:**
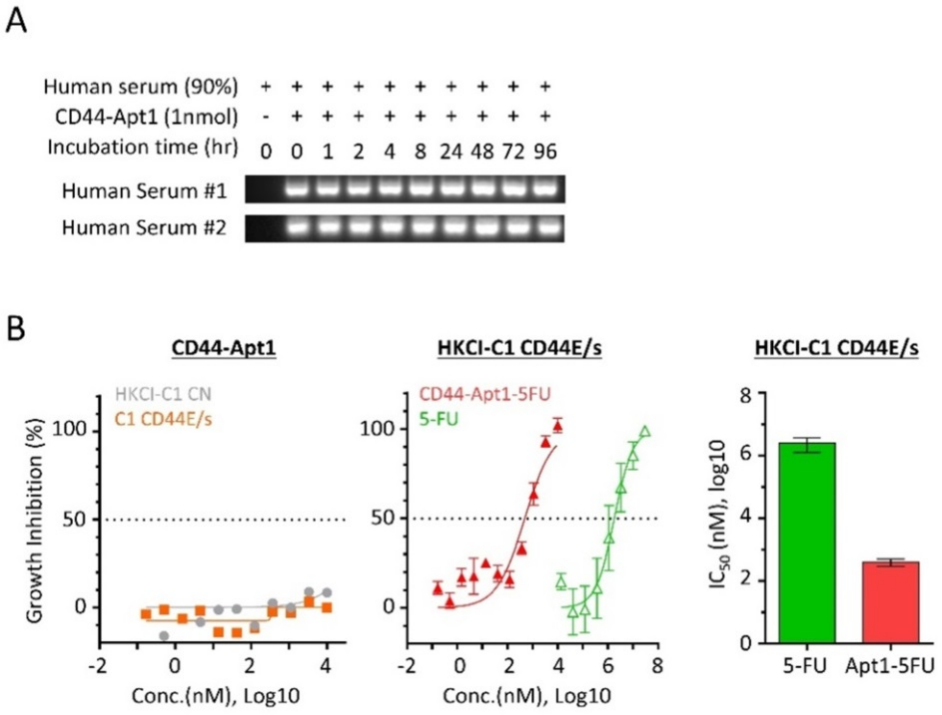
** Serum stability and drug guiding capability of CD44-Apt1. (A)** Biostability of CD44-Apt1. One nmol of phosphorothioate-modified CD44-Apt1 was incubated with human serum at a ratio of 1:9 for as long as 96 hours at 37 °C and then resolved in 3% agarose gel. **(B)** Growth inhibition effect on HCC cells by 5-FU with or without CD44-Apt1 conjugation. (**Left**) HKCI-C1 control (grey, circle) and CD44E/s cells (orange, square) were treated with concentration of 169pM to 10µM of the phosphorothioate-modified CD44-Apt1. (**Middle**) HKCI-C1 CD44E/s cells were treated with concentrations of 169 pM to 10 µM of CD44-Apt1-5FU (Red, solid triangle) or 14 µM to 30 mM of 5-FU alone (green, open triangle). (**Right**) Viability was measured using Celltiter-Glo, and growth inhibition percent was calculated. Half-maximal inhibitory concentration (IC50) was determined using the model of “log of inhibitor versus response with variable slope” under non-linear regression (mean ± SEM, N=4, each N with n=4).

Since aptamers are nucleic acids, its phosphodiester bond between nucleotides is susceptible to nuclease cleavage when exposed to biological media or *in vivo* conditions. To overcome the degradation problem, the phosphodiester bonds were modified to phosphorothioate linkage by substituting the non-bridging oxygen atoms in the phosphodiester bonds to sulfur atoms 20. The biostability of phosphorothioate-modified CD44-Apt1 was tested against nucleases in human serum (**Figure 3A**). For each time point from 0 to 96 hours, a total of one pmol of CD44-Apt1 was incubated with two freshly prepared human serums separately in a 1:9 dilution ratio in normal body temperature. After incubation, the mixtures were resolved by gel electrophoresis. Compared to the control at time 0, no degradation of CD44-Apt1 was observed across time. This indicated that the modified CD44-Apt1 could be highly stable in circulation. This phosphorothioate-modified CD44-Apt1 was then used for the following experiments on *in vitro* cell-based and *in vivo* animal assessments.

To determine if pure CD44-Apt1 exerts any cytotoxic effects, various concentrations of CD44-Apt1 were incubated with HKCI-C1 CD44E/s and control cells for 72 hours and then evaluated for cell viability (**Figure 3B**). Even at an extraordinary high concentration of 10µM, i.e. 3,000-fold of CD44-Apt1 binding affinity to CD44 proteins, more than 70% of cells were still alive after 3 days of incubation. No cell death was recorded when incubated at a moderate concentration of 1µM CD44-Apt1. This suggested CD44-Apt1 is non-toxic to living cells.

Anti-cancer drug fluoropyrimidine 5-fluorouracil (5-FU) is one of the most commonly used chemotherapeutic agents for treating malignant tumors, such as advanced HCC, and works by inducing DNA or RNA damages through misincorporation 21. In clinical practice, moderate-to-high doses of 5-FU are considered necessary to achieve satisfactory anti-tumor efficacy, although the associated deleterious side effects are also common. We tested if CD44-Apt1 could improve drug efficacy of 5-FU on HCC cells. CD44-Apt1 was conjugated with three successive 5-FU phosphonamidites at its 3' end, designated as CD44-Apt1-5FU. HKCI-C1 cells with CD44E/s overexpression were treated at various concentrations of CD44-Apt1-5FU for 3 days. When 5-FU was applied alone, CD44E/s expressing HKCI-C1 showed a 50% growth inhibition at IC50 of 2.47 ± 1.22 mM (**Figure 3B**). When delivered by CD44-Apt1, CD44E/s positive HKCI-C1 showed dramatic sensitivity to CD44-Apt1-5FU with an IC50 as low as 0.40 ± 0.11 μM. Between aptamer-deliver 5FU application and 5FU alone, a clear enhanced drug sensitivity was observed with CD44-Apt1-5FU, demonstrating a reduced IC50 value by more than 6,134 folds. Taken together with the high affinity and high sensitivity features of CD44-Apt1 to CD44E/s proteins, CD44-Apt1 could work as an efficient mediator to transport anti-cancer drugs, like 5-FU among others, into CD44E/s-expressing HCC tumor cells for enhanced intracellular drug loadings and thereof cytotoxic response. In addition, the dosage of anti-cancer drug could be vastly lowered to reduce adverse side effects in patients.

### CD44-Apt1 homing to CD44E/s-positive HCC xenografts *in vivo*

**Figure 4:**
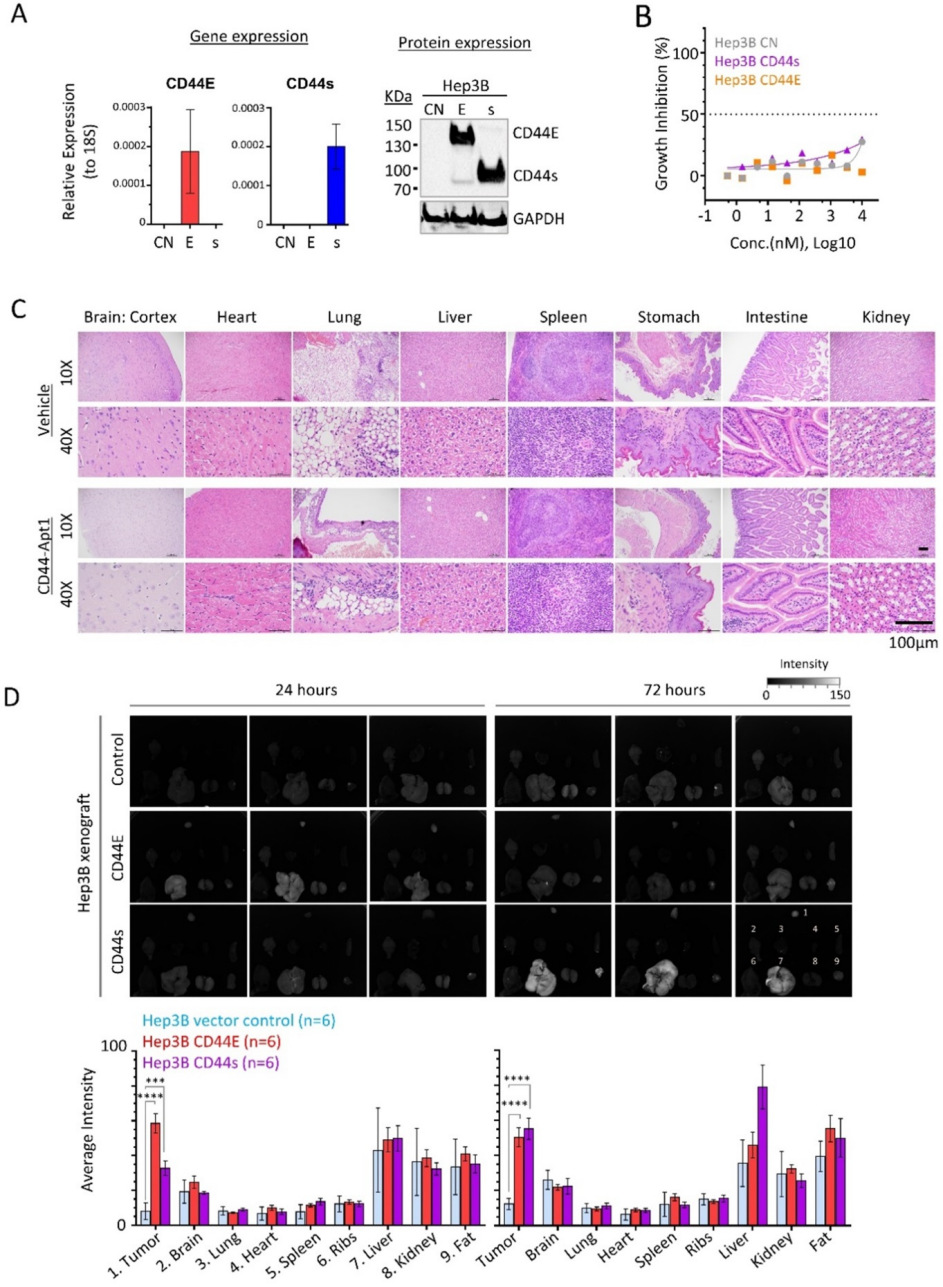
***In vivo* homing and non-toxic nature of CD44-Apt1. (A)** Establishment of CD44E- and CD44s-overexpressed Hep3B stable cell lines. **(B)** *In vitro* cytotoxicity of CD44-Apt1 on the Hep3B stable cell lines. Hep3B vector control (grey), CD44s-OE (purple), or CD44E-OE (orange) cells were incubated with various concentration of CD44-Apt1, ranging from 56 pM to 10 µM, for 72 hours. Cytotoxicity was represented in term of percentage of growth inhibition (mean ± SEM, n=3). **(C)** *In vivo* cytotoxicity. A total of 250 pmol of phosphorothioate-modified CD44-Apt1 or vehicle buffer was intravenously injected into Nude mice and incubated for 72 hours. Major organs, were harvested and processed for haemotoxylin and eosin (H&E) staining for histological imaging. **(D)** *Ex-vivo* fluorescence imaging of the HCC xenograft tumor and major organs of the aptamer-injected animals. HCC xenograft tumors were developed in Nude mice by subcutaneous injection of 5 x 10^6^ cells of Hep3B vector control, CD44E-OE, or CD44s-OE cells. When xenograft reached size of about 200mm^3^, 250 pmol of Cy3-labeled CD44-Apt1 was intravenously injected into tumor-bearing mice and incubated for 24 or 72 hours before sacrifice. Fluorescence intensity was measured in the tumor and organs and compared between xenograft models using 2-way ANOVA test (mean ± SD, n=6). Two independent experiments were carried out with 3 mice per group. Another set of images were displayed in Supplementary Figure 2. **p <0.01, ***p<0.001.

Efficient and specific delivery of aptamer-based therapeutics *in vivo* is one the major concerns in developing targeted therapy. Since Hep3B xenograft could be efficiently developed, we generated two stable cell lines with overexpression of either CD44E or CD44s (**Figure 4A**). Once again, CD44-Apt1 did not induce cell death in all three Hep3B isogenic lines, including the parental and two CD44 variants overexpressing cells (**Figure 4B**).

We then tested the *in vivo* cytotoxicity of CD44-Apt1. A total of 250 pmol of either CD44-Apt1 or vehicle was intravenously injected into nude mice and incubated for 3 days. Major organs, including brain, heart, lung, liver, spleen, stomach, intestine, and kidney were obtained and examined for histological differences (**Figure 4C**). Histological staining showed that all harvested organs looked intact with no apparent tissue damage. This result further supported the non-toxic nature of CD44-Apt1 at the tested conditions.

Next, we determined the *in vivo* homing capability of CD44-Apt1 to CD44E or CD44s positive Hep3B xenografts. Cy3-conjugated CD44-Apt1 at 250pmol was intravenously injected into mice bearing xenografts that were developed by subcutaneously injection of Hep3B CD44E, CD44s, or parental cells. After 24 or 72 hours of incubation, tumor and major organs were harvested and evaluated for Cy3 signal. Since strong autofluorescence was detected in stomach and intestine, analysis of accumulation of CD44-Apt1 were ignored in both organs (Supplementary Figure 1). Significantly accumulation of Cy3 signal was readily observed in CD44E xenografts at 24 hours and maintained at high level after 72 hours of incubation, while its levels in control xenografts were barely detectable at both time points (**Figure 4D**). In CD44s xenografts, CD44-Apt1 accumulation was 3-fold higher than that in control xenografts after 24 hours although a statistical value was not achieved. It subsequently elevated to a significant level at 72 hours. The steady accumulation of CD44-Apt1 in CD44s xenografts is suggestive of phosphorothioate-modified CD44-Apt1 could prolong half-life of aptamers under *in vivo* physiological conditions, although adversely this has also caused retention in the liver and kidney. Detection of Cy3 signals in other major organs, such as ribs, hearts, spleens, and lungs were nonetheless low.

**Figure 5:**
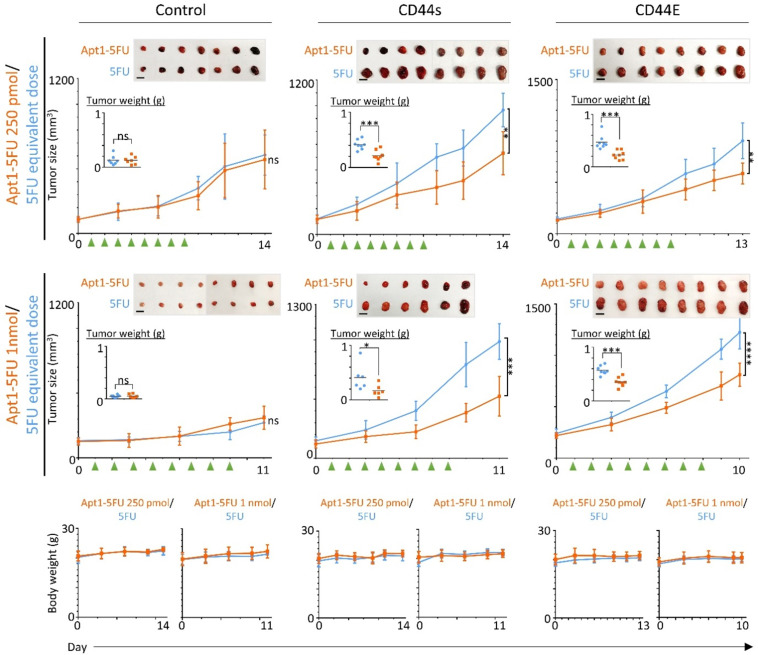
***In vivo* growth inhibition effect on HCC cells by 5-FU with or without CD44-Apt1conjugation.** Subcutaneous HCC xenograft tumors were developed using Hep3B vector-control, CD44E-OE, or CD44s-OE cells. When xenograft reached size of about 100mm^3^, 250 pmol or 1nmol of CD44-Apt1-5FU (orange, square), or equivalent dosage of 5-FU (blue, circle) was intravenously injected for 8 consecutive days into tumor-bearing mice. Green arrows indicated the day of injection. The tumors were allowed to grow for 2 to 6 days before sacrifice. Tumor size and body weight were measured every two or every other day and compared between the two treatment groups using 2-way ANOVA test (mean ± SD). Tumors were weighted at the end point and compared between the two treatment groups using unpaired t-test. *p <0.05, **p <0.01, ***p<0.001, **** p<0.0001.

Finally, we tested the *in vivo* anti-cancer drug delivery ability of CD44-Apt1 for promoting growth inhibition of CD44E or CD44s positive Hep3B xenografts in Nude mice. CD44-Apt1-5FU or 5FU were intravenously injected into the mice bearing Hep3B vector control, CD44s, or CD44E xenografts for eight consecutive days (Figure 5). The CD44s and CD44E tumors but not the vector-control tumors were significantly reduced in size across time and in weight at the endpoint after being treated with the CD44-Apt1-5FU compared to those treated with 5FU. Since the body weight of the treated mice was stably increasing, no sign of adverse effects on the health of the treated mice was suggested with the current treatment schedule and dosings.

Taken together, CD44-Apt1 could home to CD44E/s-expressing HCC xenografts and could be a guiding probe in therapy.

## Discussion

CD44 is the principal cell surface receptor that mediates cell adhesion and governs multiple cellular functions 9, 10. Alternative splicing of CD44 have been reported to generate a number exon-skipping CD44 variants. Many of these have been further shown to play critical roles in oncogenesis and metastatic spread during tumor progression 11-14. Our study showed common overexpression of CD44E and/or CD44s in as much as 61.1% of primary HCC tumors, with functions of CD44E/s in promoting HCC cell proliferation and migration. These tumor enriched CD44 variants represent ideal cell-surface markers for aptamer nanoprobe development that can specifically target HCC cells.

To our knowledge, three CD44 aptamers had been reported in the literature. The first is a DNA aptamer with mono-thiophosphate modification targeting the HABD region of CD44 and was identified using *in vitro* SELEX using *E. coli* synthetic recombinant protein of the HABD 22. It was demonstrated to bind several ovarian cancer cell lines 22 and an age-related macular degeneration model 23 with a binding affinity with K_D_ of 181nM_._ A recent study showed that this CD44 aptamer alone could reduce SNAIL expression and migratory property of a breast cancer cell line 24. The second CD44 aptamer is a 2'-F-pyrimidine modified RNA aptamer, which was selected by *in vitro* SELEX using mammalian system-derived full-length synthetic CD44 protein as target 25. Exact epitope of this aptamer is unknown. This CD44 RNA-aptamer could bind breast cancer cell lines, and its binding affinity to recombinant CD44 protein is at K_D_ of 81nM 25. This RNA-aptamer was shown to trigger apoptosis of some CD44-enriched ovarian cancer cell lines* in vitro*, and suppressed tumor peritoneal metastasis in OVAR8 xenograft model *in vivo* 26. In another study, gefitinib-loaded nanomicelles with conjugation of this CD44 RNA aptamer inhibited spheroid formation of CD133-positive lung cancer cells 27. The third published CD44 aptamer targeted also the HABD recombinant protein 28. This CD44 DNA aptamer, named s5rev, exhibited a K_D_ of 238 nM with cytotoxic effect shown on a leukemic cell line NB4, possibly via inhibiting the Akt pathway 28.

In this study, we employed the loss-gain cell-SELEX method to discover CD44E/s targeting aptamers. Among four identified aptamers, CD44-Apt1 showed superior binding affinity with a K_D_ as low as ~1nM, which is the highest affinity of any reported CD44 aptamers. We further defined the epitope of CD44-Apt1 to be part of the glycosylation site-enriched variable domain, which is encoded by Exons 15 to 16. Unlike the two reported CD44-HABD aptamers, CD44-Apt1 does not bind to the HABD region and is specific to CD44E/s presence. It exhibits strong penetrative ability by efficiently localizing into nuclei. Similar to many aptamers, CD44-Apt1 is non-cytotoxic as demonstrated in our *in vitro* cell-based assay and *in vivo* animal test. Phosphorothioate-modified CD44-Apt1 displayed extraordinary stability in serum, which in turn facilitated the efficient homing of CD44-Apt1 to CD44-positive HCC xenograft *in vivo*.

In this study, CD44E/s-aptamer delivered 5-FU treatment dramatically increased drug cytotoxicity by more than 6,134-folds in HCC cells via the CD44E/s-mediated endocytosis. To overcome the problem of *in vivo* retentions in liver and kidney, future studies would require adjustment of CD44-Apt1 backbone for phosphorothioate modifications in balancing half-life *in vivo* and durability for intracellular drug loading. Nevertheless, our findings both *in vitro* and *in vivo* concurred in highlighting the great potential of CD44E/s aptamers as a drug guiding probe. Other than by oligo synthesis-based conjugation method, as shown in linking aptamers with 5-FU in our study, CD44E/s aptamers could also be loaded with other therapeutic drugs, for instance sorafenib which is the first-line FDA approved drug for HCC, by methods such as liposome-based nanocarriers 29. It could also be linked with siRNA/miRNA for targeted gene therapy.

In conclusion, our discovered CD44-Apt1 shows high sensitivity and affinity as a CD44E/s dual-targeting DNA aptamer. The high cell-penetrating capability and drug guiding ability of CD44-Apt1 could serve as a useful tool to efficiently arrest CD44E/s-positive HCC tumors through guiding therapies.

## Figures and Tables

**Table 1:** Sequences of identified CD44E/s aptamers

Aptamer ID	Sequence
**CD44E-Apt01**	**ATCCAGAGTGACGCAGCATCGCAACGATTAGTATGCACCCACCGTATAGGTTGGTCTCTGGACACGGTGGCTTAGT**
CD44E-Apt03	ATCCAGAGTGACGCAGCACATATAACGAAGAACCAGAACGGAAGGGAACCTCTGCGTGTGGACACGGTGGCTTAGT
CD44E-Apt04	ATCCAGAGTGACGCAGCAACAGGGGAAGCATCTATAGTCAGGACCCGTCTGATCACACTGGACACGGTGGCTTAGT
CD44E-Apt05	ATCCAGAGTGACGCAGCACCAGGGAAGCATCTATAGTCAGGACCTGGAAGGAGCGTTATGGACACGGTGGCTTAGT

## Abbreviations

CD44E: CD44v8-10
HCC: Hepatocellular carcinoma
5-FU: 5-flurorouracil
cell-SELEX: Cell-based systematic evolution of ligands by exponential enrichment
HABD: Hyaluronic acid binding domain
IP3 receptor: Inositol 1,4,5-triphosphate receptor
MMP: Matrix metalloproteinases
ROS: Reactive oxygen species
EMT: Epithelial to mesenchymal transition
