# Supplementary material for: Development of CD44E/s dual-targeting DNA aptamer as nanoprobe to deliver treatment in hepatocellular carcinoma
Source: Nanotheranostics. 2022 Jan 1;6(2):161–74. doi: 10.7150/ntno.62639 (PMC8671951; doi:10.7150/ntno.62639)
Supplement: Supplementary file 1 — Supplementary figure S1. [file ntnov06p0161s1.pdf]

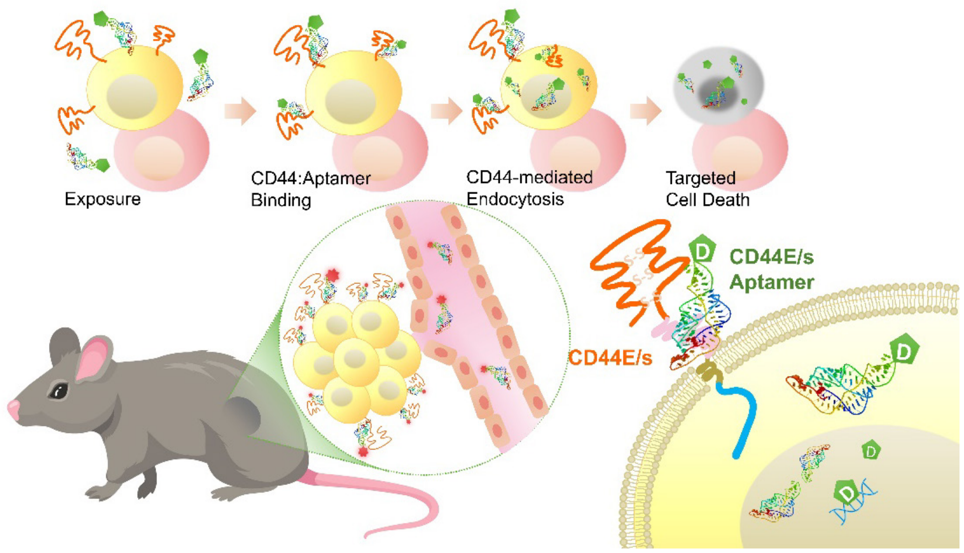

Supplementary Figure S1. A CD44E/s dual-targeted aptamer specifically arrests CD44E/s-positive tumor *in vivo* and efficiently delivers drug into CD44E/s-positive cells resulting in dramatic increase in intracellular drug loading and consequent targeted cell death. (*29 words*)
